# Ability of ELISAs to detect antibodies against porcine respiratory and reproductive syndrome virus in serum of pigs after inactivated vaccination and subsequent challenge

**DOI:** 10.1186/s12917-016-0888-0

**Published:** 2016-11-21

**Authors:** Tatjana Sattler, Jutta Pikalo, Eveline Wodak, Friedrich Schmoll

**Affiliations:** 1 Large Animal Clinic for Internal Medicine, University of Leipzig, An den Tierkliniken 11, 04103 Leipzig, Germany; 2 Institute for Veterinary Disease Control, AGES, Robert-Koch-Gasse 17, 2340 Mödling, Austria

**Keywords:** Swine, ELISA, PRRSV, Inactivated vaccine

## Abstract

**Background:**

In this study, six enzyme-linked immunosorbent assays (ELISA), intended for routine porcine reproductive and respiratory syndrome virus (PRRSV) herd monitoring, are tested for their ability to detect PRRSV specific antibodies in the serum of pigs after vaccination with an inactivated PRRSV type 1 vaccine and subsequent infection with a highly pathogenic (HP) PRRSV field strain. For this reason, ten piglets (group V) from a PRRSV negative herd were vaccinated twice at the age of 2 and 4 weeks with an inactivated PRRSV vaccine. Ten additional piglets (group N) from the same herd remained unvaccinated. Three weeks after second vaccination, each of the piglets received an intradermal application of an HP PRRSV field strain. Serum samples were taken before first vaccination as well as before and 3, 7, 10 and 14 days after HP PRRSV application. All serum samples were tested for PRRSV RNA by reverse transcriptase quantitative polymerase chain reaction (RT-qPCR) as well as for PRRSV antibodies with all six study ELISAs.

**Results:**

At the beginning of the study (before vaccination), all of the piglets were PRRSV antibody negative with all study ELISAs. They also tested negative for PRRSV RNA measured by RT-qPCR. From day 3 after HP PRRSV application until the end of the study, a viremia was detected by RT-qPCR in all of the piglets. On day 0 (day of HP PRRSV application), nine out of ten piglets of the pre-vaccinated group tested PRRSV antibody positive with one of the tested ELISAs, although with lower S/P values than after infection. On day 10 after HP PRRSV application, all study ELISAs except one had significantly higher S/P or OD values, respectively more positive samples, in group V than in group N.

**Conclusions:**

Only one of the tested ELISAs was able to detect reliably PRRSV antibodies in pigs vaccinated with an inactivated PRRSV vaccine. With most of the tested ELISAs, higher S/P values respectively more positive samples after PRRSV infection were seen in the pre-vaccinated group than in the non-vaccinated.

## Background

In studies validating or comparing new enzyme-linked immunosorbent assays (ELISA) for the detection of antibodies (Ab) against the porcine reproductive and respiratory syndrome virus (PRRSV), the IDEXX PRRS X3 Ab test (IDEXX, Westbrook, USA) is usually used as the gold standard [1–4]. This ELISA system has a sensitivity of 98.8% and a specificity of 99.9%, according to manufacturer. In one study using serum samples collected from challenged pigs, a sensitivity of 100% was found [5]. Measured with the IDEXX ELISA, an antibody response can be detected beginning between days 9 and 12 after PRRSV infection or vaccination with attenuated PRRSV live vaccines [2, 6–9] and lasting at least until day 120 after vaccination or challenge [10]. Although the application of certain inactivated PRRSV vaccines seems to prime the immune system and can, according to some studies, lead to a faster and more effective immune response after PRRSV infection [11, 12], no antibody response has been observed with the IDEXX ELISA after vaccination with inactivated PRRSV vaccines [10, 13]. Therefore the question arises as to whether or not other ELISAs are capable of detecting PRRSV specific Ab in the serum of pigs vaccinated with an inactivated PRRSV vaccine. Studies conducted with a blocking ELISA [2] and a commercial ELISA [14] hint towards this possibility. In a study published by Cong et al. [2], a combination of a nucleocapsid based ELISAs and an ELISA based on non-structural proteins (NSP) as antigens was even able to differentiate inactivated vaccine-derived Ab from Ab evoked by live vaccines. The ELISAs described in the study, however, are not commercially available. For routine monitoring, new versions of commercial ELISAs as well as some recently developed ELISAs, based on the nucleocapsid or other antigens are available or will soon be on the market.

The objective of the presented study was to evaluate if commercial and/or newly developed PRRSV Ab ELISAs are able to detect PRRSV Ab in the serum of pigs after vaccination with an inactivated PRRSV type 1 vaccine. Furthermore, the influence of the inactivated vaccination on Ab development, measured with the respective ELISAs, after subsequent intradermal application of a highly pathogenic (HP) PRRSV field strain was compared with a non-vaccinated group. Differences between the ELISAs are emphasised.

## Methods

### Study design, animals and serum samples

The study included 20 piglets from a PRRSV free farm. Ten piglets (group V) were pre-vaccinated twice at the age of 2 and 4 weeks (days -33 and -19 of the study) with an inactivated PRRSV vaccine in an oil-and-water adjuvant (2 ml i.m., Progressis, Merial, Batch No. L408629). The other 10 piglets (group N) were not pre-vaccinated. All 20 piglets received an intradermal application of 0.2 ml of a HP PRRSV field strain (Vietnam_PRRSV_AGES/568-30FC/13; GenBank accession number KM588915) diluted in an adjuvant (Diluvac Forte, Intervet, Unterschleissheim, Germany). Serum samples were taken directly before and 33 days after first vaccination (=day 0 of the study, directly before HP PRRSV application) as well as 3, 7, 10 and 14 days after HP PRRSV application.

### Molecular analysis

All serum samples were tested on each sampling point with a commercial reverse transcriptase quantitative polymerase chain reaction (RT-qPCR) assay (TaqMan® NA and EU PRRSV Reagents and Controls, ThermoFisher Scientific, Vienna, Austria) for the presence of PRRSV RNA as described previously [1].

### Detection of PRRSV Ab by ELISA

The serum samples of all of the piglets at each sampling point were tested for Ab against PRRSV by the following ELISAs: a) IDEXX PRRS X3 Ab test – in the following called IDEXX, b) INgezim PRRS 2.0 (Ingenasa, Madrid, Spain) – INgezim, c) Civtest suis PRRS A/S plus (Laboratorios HIPRA, Amer, Spain) – HIPRA A/S, d) Civtest suis PRRS E/S plus (Laboratorios HIPRA) – HIPRA E/S, e) pigtype® PRRSV Ab (QIAGEN, Leipzig, Germany) – QIAGEN, f) PRRSV CHECK ELISA (Analytik Jena, aj Roboscreen, Leipzig, Germany) – AJ.

All ELISAs were performed according to manufacturer’s instructions.

### Statistical analysis

The results of the ELISAs measured in the serum samples were described as positive or negative for PRRSV Ab. Differences between the outcomes of the ELISAs at each time point as well as differences between the groups for each ELISA on each time point were calculated with the Fisher’s exact test. The sensitivity of the ELISAs at each sampling point and the 95% confidence interval using the method of Clopper and Pearson were determined. The S/P or OD values for each sampling point were shown in boxplots using median, quartiles and 95% confidence interval. S/P and OD values at each sampling point were tested for normal distribution using the Kolmogorow-Smirnov test. Since most parameters were not normally distributed, differences between of the S/P or OD values in both groups at each sampling point were determined with the Mann–Whitney test. Differences between the S/P values of the ELISAs on the sampling points before and after were tested using the Friedman test followed by the Wilcoxon test as post hoc test. A Bonferroni correction of the error of probability was done. Differences with *P* < 0.05 were considered significant.

## Results

### Molecular analysis

Before vaccination with the inactivated vaccine in group V as well as on day 0 (day of HP PRRSV application), all of the serum samples from the piglets from both groups tested negative by PRRSV RT-qPCR. On day 3 after HP PRRSV application until the end of the study, all of the piglets in the study tested positive by PRRSV RT-qPCR as it was expected.

### Detection of PRRSV antibodies by ELISA

**Fig. 1:**
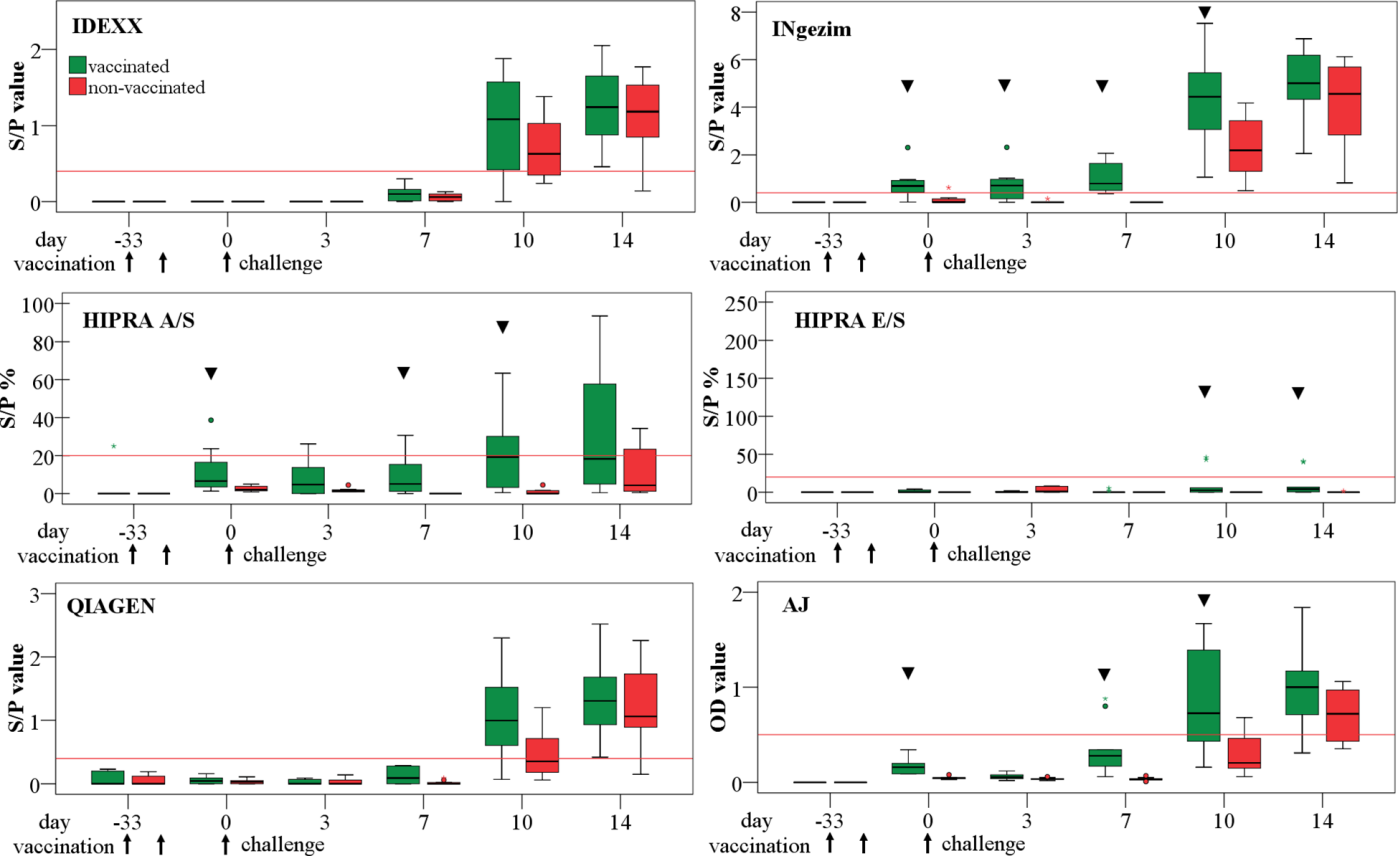
Boxplots of S/P values, respectively OD values of all tested PRRSV Ab ELISAs. Vaccinated group: vaccination with an inactivated PRRSV type 1 vaccine on days -33 and -19 (see *arrows*), both groups: intradermal application of an HP PRRSV field strain on day 0 (see *arrows*). *Red lines*: cut-off of the ELISAs. The *black triangle* indicates significant differences between the groups

At the beginning of the study (day -33), serum samples from all of the piglets from both groups tested negative in all of the study ELISAs. Tested by the INgezim ELISA, nine out of ten piglets of group V and one out of ten piglets of group N were PRRSV Ab positive on day 0. The number of PRRSV Ab positive piglets at each sampling point is given in Table 1. The piglets from group V remained Ab positive until day 14 with the INgezim ELISA with significantly higher S/P values on days 10 and 14 than on days 0 and 3. Significant differences between the ELISAs regarding the number of positive piglets at each sampling day are indicated in Table 1. The sensitivity of the ELISAs at each time point can be seen in Table 2. Figure 1 shows the S/P and OD values of each ELISA at the different sampling points. Measured with INgezim ELISA, the S/P values in group V were significantly higher than in group N on days 0, 3, 7, and 10. With the HIPRA A/S, group V had significantly higher S/P values than group N on days 0, 7 and 10, although fewer samples were Ab positive than in the other ELISAs. Significantly higher S/P values in group V on days 10 and 14 were also found with the HIPRA E/S with two positive piglets on both sampling points. With the AJ ELISA, significant differences between the groups were seen on days 0, 7 and 10 with higher S/P values in group V resulting in two positive samples in group V on day 7. Significantly more piglets from group V were positive on day 10, tested with the HIPRA A/S and the AJ ELISA than in group N. The IDEXX and QIAGEN ELISAs showed no significant differences in S/P values between group V and N, although a tendency in higher S/P values in group V was seen in the QIAGEN ELISA on day 10 (Fig. 1) with a tendency of more positive samples in group V than in group N (Table 1).

**Table 1:** Results of PRRSV Ab ELISAs at the sampling points, number of positive animals

Study day	-33	0	3	7	10	14
	*n* = 10	*n* = 10	*n* = 10	*n* = 10	*n* = 10	*n* = 10
Group V						
IDEXX	0	0^b^	0^b^	0^b^	8^a,b^	10^a^
INgezim	0	9^a^	7^a^	10^a^	10^a^	10^a^
HIPRA A/S	0	2^b^	2^b^	1^b^	5^b^	5^b^
HIPRA E/S	0	0^b^	0^b^	0^b^	2^c^	2^c^
QIAGEN	0	0^b^	0^b^	0^b^	8^a,b^	10^a^
AJ	0	0^b^	0^b^	2^b^	7^a,b^	8^a,b^
Group N						
IDEXX	0	0	0	0	7^b,c^	9^a^
INgezim	0	1	0	0	10^a,b^	10^a^
HIPRA A/S	0	0	0	0	0^c^	3^b,c^
HIPRA E/S	0	0	0	0	0^c^	0^c^
QIAGEN	0	0	0	0	4^c^	9^a^
AJ	0	0	0	0	1^c^	6^a,b^

Group V: vaccination with an inactivated PRRSV type 1 vaccine on days -33 and -19

Groups V and N: intradermal application of an HP PRRSV field strain on day 0

Different letters indicate significant differences between the ELISAs in each group on each sampling point

**Table 2:** Sensitivity (%) of PRRSV Ab ELISAs at each sampling point (95% confidence interval)

Study day	0	3	7	10	14
Group V					
IDEXX	0 (0–30)	0 (0–30)	0 (0–30)	80 (44–98)	100 (69–100)
INgezim	90 (60–98)	70 (35–93)	100 (69–100)	100 (69–100)	100 (69–100)
HIPRA A/S	20 (3–56)	20 (3–56)	10 (0–45)	50 (19–81)	50 (19–81)
HIPRA E/S	0 (0–30)	0 (0–30)	0 (0–30)	20 (3–56)	20 (3–56)
QIAGEN	0 (0–30)	0 (0–30)	0 (0–30)	80 (44–98)	100 (69–100)
AJ	0 (0–30)	0 (0–30)	20 (3–56)	70 (35–93)	80 (44–98)
Group N					
IDEXX	n.d.	0 (0–30)	0 (0–30)	70 (35–93)	90 (60–98)
INgezim	n.d.	0 (0–30)	0 (0–30)	100 (69–100)	100 (69–100)
HIPRA A/S	n.d.	0 (0–30)	0 (0–30)	0 (0–0)	30 (7–65)
HIPRA E/S	n.d.	0 (0–30)	0 (0–30)	0 (0–0)	0 (0–0)
QIAGEN	n.d.	0 (0–30)	0 (0–30)	40 (12–74)	90 (60–98)
AJ	n.d.	0 (0–30)	0 (0–30)	10 (0–45)	60 (26–88)

Group V: vaccination with an inactivated PRRSV type 1 vaccine on days -33 and -19

Groups V and N: intradermal application of an HP PRRSV field strain on day 0

*n.d.* not done

## Discussion

This study tested the ability of six ELISAs for detection of PRRSV Ab after inactivated vaccination of piglets and subsequent infection with HP PRRSV. Data are published about the performance, sensitivity and specificity of the ELISAs tested in this study [1, 6, 15, 16]. They did, however, not concern serum of pigs vaccinated with an inactivated vaccine. Therefore, this aspect was illuminated in this study.

The nucleocapsid-based IDEXX PRRS X3 Ab test is usually used as the gold standard for detection of PRRSV Ab by ELISA [1–4]. In our study, with this ELISA, however, no Ab could be detected in serum samples of piglets vaccinated with an inactivated vaccine. This confirms the findings of another study, where no antibody response was detected with the IDEXX ELISA after inactivated PRRSV vaccination as well [10]. As expected from the results of other studies [2, 6, 16], the IDEXX ELISA tested most piglets PRRSV Ab positive from day 10 after infection onwards. According to a study by Zuckermann et al., the IDEXX ELISA was able to detect higher S/P values in pigs pre-vaccinated with an inactivated PRRSV vaccine than in non-vaccinated pigs on days 7 and 10 after the subsequent challenge [13]. In our study, however, no differences were found between vaccinated and non-vaccinated piglets with the IDEXX ELISA. In contrast to the IDEXX ELISA, some of the other tested ELISAs were able to detect PRRSV Ab in piglets vaccinated with the inactivated vaccine. An especially high sensitivity in this respect was found in the similarly nucleocapsid-based INgezim ELISA that tested most of the samples from the vaccinated piglets PRRSV Ab positive. This confirms the results of a previously published study that describes the ability of a former version of an ELISA produced by Ingenasa to detect PRRSV Ab after inactivated vaccination of pigs [14]. The S/P values seen in the INgezim ELISA after inactivated vaccination were significantly lower than on days 10 and 14 after HP PRRSV application. Low PRRSV Ab S/P values detected with the INgezim ELISA in groups of piglets from PRRSV negative farms can be a sign for inactivated PRRSV vaccination, especially when no antibodies can be found with the IDEXX or another of the tested ELISAs. A reliable differentiation between vaccinated and infected pigs, however, is not possible. One positive result was found with the INgezim ELISA in the non-vaccinated group at day 0. The specificity of this ELISA was calculated with 99% in another study, which is slightly lower than the specificity of the IDEXX ELISA [1]. This can be the explanation for the false positive result. The specificities of the other tested ELISAs have been calculated in the mentioned study as well [1].

With the HIPRA A/S ELISA that is based on membrane glycoproteins and in the AJ ELISA, based on a mixture of structural proteins, including the nucleocapsid, on day 10 significantly more samples were tested positive in group V than in group N. In tendency, more vaccinated piglets were Ab positive on day 10 tested with the QIAGEN ELISA. This would, however, not allow a differentiation between vaccinated and non-vaccinated pigs after PRRSV infection, since the ELISAs are not quantitative, but give only qualitative results.

With the INgezim ELISA, significantly more piglets were tested positive from days 3 to day 14 in group V and on days 10 and 14 in group N than with most of the other ELISAs which goes confirm with the calculated sensitivity of this ELISA. The sensitivity of the ELISAs at each time point was highest in the nucleocapsid based ELISAs, especially in the INgezim ELISA. Lower sensitivities were found with the HIPRA ELISAs and the AJ ELISA. For both of them, a later onset of Ab detection than for the nucleocapsid based ELISAs has been described in another study and is due to the antigen used in the ELISAs [16].

The adding of an adjuvant and the intradermal administration route of the HP PRRSV virus was used to induce a strong Ab response detectable by the ELISAs. An adjuvant as it was used for the challenge strain in this study is able to enhance Ab production to a certain degree without a vaccine [10]. Intradermal application of a modified live vaccine was shown to elicit an effective immune response [17].

It cannot be concluded from the results of our study if the development of a protective immunity after infection would be enhanced in the vaccinated piglets. Some studies are available that refer to protective immune response after vaccination against PRRSV with a novel killed vaccine [11, 12]. Another study used a killed PRRSV vaccine in previously infected pigs which resulted in increased serum neutralization Ab titers and interferon γ producing cells, although the virus shedding was not affected [18]. Other studies did not show killed vaccines to have any protective effect including the one used in this study [2, 14].

The inactivated PRRS vaccine used in group V belongs to the same subgroup of type 1 as the Lelystad strain with 99% homology to this strain [11]. This was confirmed in our laboratory as well (data not shown). Although the HIPRA A/S ELISA claims to detect PRRSV type 2 antibodies exclusively, the S/P values in serum of the pigs from group V treated with an inactivated PRRSV type 1 vaccine were significantly higher than in the non-vaccinated piglets. The HIPRA E/S, on the other hand, reacted only slightly. This indicates some cross reactions between PRRSV type 1 and 2 Ab, detected with the HIPRA ELISAs. These cross reactions were seen in another study as well [16] and are also acknowledged by the manufacturer.

## Conclusions

This study showed that, among the tested ELISAs, only the INgezim ELISA was able to detect an Ab development in most of the piglets after inactivated PRRS vaccination and before the infection with HP PRRSV field strain, although with lower S/P values than after the infection. This ELISA can therefore be used to detect Ab in PRRSV negative pigs vaccinated with an inactivated PRRS vaccine. Five of the six tested ELISAs, based on nucleocapsid, such as the INgezim and QIAGEN ELISAs, or other antigens, as in the HIPRA and AJ ELISAs, are able to show a difference in Ab development between piglets vaccinated with an inactivated vaccine and subsequent HP PRRSV application and piglet not pre-vaccinated before HP PRRSV application. These differences were not seen when using the IDEXX ELISA. The HIPRA A/S plus ELISA is able to detect exclusively PRRSV type 2 Ab, although some cross reactions with PRRSV type 1 Ab are expected. The different characteristics and performance of the available test systems have to be considered when choosing an ELISA for PRRSV monitoring in pig herds.

## Abbreviations

Ab: Antibodies
ELISA: Enzyme-linked immunosorbent assay
HP: Highly pathogenic
NSP: Non-structural proteins
PRRSV: Porcine reproductive and respiratory syndrome virus
RT-qPCR: Reverse transcriptase quantitative polymerase chain reaction
